# Cholinergic Phenotypes of Acetyl-CoA with ATP-Citrate Lyase Link

**DOI:** 10.3390/ijms27020782

**Published:** 2026-01-13

**Authors:** Sylwia Gul-Hinc, Agnieszka Jankowska-Kulawy, Andrzej Szutowicz

**Affiliations:** Department of Laboratory Medicine, Chair of Clinical Biochemistry, Medical University of Gdańsk, 80-210 Gdansk, Poland; aja@gumed.edu.pl

**Keywords:** cholinergic neuron, ATP-citrate lyase, choline acetyltransferase, pyruvate dehydrogenase, acetylcholine, metabolic compartmentation

## Abstract

Glycolysis-derived pyruvate is the almost exclusive source of acetyl-CoA for energy production in mitochondrial compartments of all types of neuronal and glial cells. Neurons utilize several times more glucose than glial cells due to their neurotransmitter functions. Cholinergic neurons that are responsible for cognitive functions require additional amounts of acetyl-CoA for acetylcholine-transmitter synthesis in their cytoplasmic compartment. It may be assured by preferential localization of ATP-citrate lyase (ACLY) in the cytoplasm of cholinergic neurons’ perikaryons and axonal terminals. This thesis is supported by the existence of strong regional and developmental correlations of ATP-citrate lyase and choline acetyltransferase (ChAT) activities and ACh levels in the brain. Electrolytic or chemical lesions of cholinergic nuclei cause proportional loss of the above parameters in the respective cortical target areas. On the other hand, the regional activity of mitochondrial pyruvate dehydrogenase complex (PDHC), which synthesizes nearly the whole pool of neuronal acetyl-CoA, displays no correlation with cholinergic innervation. It makes cholinergic neurons highly susceptible to brain pathologies impairing energy metabolism. Therefore, the ACLY pathway, which provides acetyl units directly to the site of acetylcholine synthesis in cholinergic nerve terminals, plays a key role in the maintenance of cholinergic neurotransmission. On the other hand, in cholinergic motor neurons, various ACLY–protein complexes are involved not only in neurotransmission but also in axonal transport of cholinergic elements from the perikaryon to cholinergic neuro-muscular junctions. This review presents findings supporting this thesis.

## 1. Introduction

The brain is the most sophisticated body organ in terms of diverse morphology, metabolic and functional multilevel intra and intercellular compartmentation and intrinsic regulatory interactions. Several reports demonstrate marked differences in levels of energy metabolism of multiple classes of neurons, micro, astro and oligodendroglial cells as well as abundant vascular epithelium constituting the blood–brain barrier. Therefore, pathologic alterations in particular cell groups in various brain regions may present a wide range of encephalopathic clinical phenotypes.

Neurons constitute about 30%, whereas microglia, astroglia and oligodendroglia form about 30, 20 and 10% of all brain cell populations, respectively [1]. These proportions may vary significantly depending on specific brain structure or region. There are also several groups of neurons synthesizing and releasing different neurotransmitters from their synaptic terminals and axonal varicosities, which either activate or inhibit respective postsynaptic dendritic spines on postsynaptic neurons. The glutamatergic and GABA-ergic neurons form the most abundant regulatory network of about 50 and 25% overall brain synapses with L-glutamate and gamma-aminobutyrate as neurotransmitters, respectively [2,3]. Note that each human neuron possesses about 10,000, whereas rats have about 2000 synapses/connections with other neurons or glial cells [1]. The density of synaptic connections exponentially increases cognitive capacities of human brains against animal ones. On the other hand, cholinergic motor neurons must convey a depolarization wave of voluntary signals from perikaryons in the brain stem and spinal cord to remote neuro-muscular junctions. They possess mechanisms for axonal transport of cholinergic enzymes and structural elements along the microtubular system, to cope with highly variable striated muscle voluntary activities.

Neuron-evoked neurotransmission is a basic function of the brain, which requires large amounts of energy. It results from the fact that neuronal signaling is linked with current depolarization–repolarization cycles of 3–50 Hz frequency. They are linked with a transmembrane against gradient relocation of Na^+^/K^+^ and Ca^2+^ cations between extra and intracellular compartments, to establish resting/action membrane potentials. To meet this demand, neurons utilize about 10 times more glucose and oxygen than other nonneuronal cells. As a consequence, in resting conditions, a brain that constitutes 2% of body mass consumes 20% of total glucose and oxygen supply, two thirds of which is utilized by neurons [4].

These properties make neurons particularly susceptible to pathologic conditions such as hypoxia, hypoglycemia, excitotoxic signaling, free radicals overload or diverse endogenous and xenobiotic cytotoxins. Such conditions upset cytoplasmic glycolysis and pyruvate production yielding limitation of its conversion to acetyl-CoA by mitochondrial pyruvate dehydrogenase complex (PDHC) [5,6]. Cholinergic neurons that utilize an additional pool of acetyl-CoA for ACh synthesis compete with other pathways for this metabolite with other synthetic pathways. Therefore, they are particularly susceptible to neurodegeneration constituting the most prevalent group of cholinergic encephalopathies in human populations [6].

## 2. Brain Cholinergic Neurons

Brain cholinergic neurons constitute a small, about 5% fraction of the overall brain neuron population, but their impact on conscience and behavior remains unconditional [7]. Their unique property is synthesis, vesicular storage and release of neurotransmitter acetylcholine (ACh). Perikaryons of cholinergic neurons responsible for cognitive functions are located in nuclei of the medial septum, diagonal band of Broca and nucleus basalis of Meynert [8]. They form axonal projections forming synaptic connections with neurons in the hippocampus and all cortical regions responsible for memory formation and all multiple cognitive functions. Their regional abundance may be assessed directly by immunohistochemical staining or indirectly by determination of activity/level of specific cholinergic markers such as choline acetyltransferase (ChAT), vesicular ACh transporter (VAChT), high-affinity choline transporter (ChT1), and muscarinic M2 autoreceptor [8,9]. Functional competence of cholinergic synapses may be assessed by testing levels of ACh accumulation and rates of its release from whole tissue, isolated cells of nerve terminal preparations or by electrophysiological methods [10,11]. These parameters distinguish well cholinergic neurons’ bodies, axons and terminals from noncholinergic ones and other cellular structures of the brain.

### 2.1. Regional and Developmental Patterns of Cholinergic Neurons

Fractional content of cholinergic neurons/terminals may vary from 30 to about 1% of the overall neuronal population in the striatum and cerebellum, respectively [12,13]. Accordingly specific activities/levels of ChAT in hippocampus, cerebral cortex and cerebellum are 2, 3 and 14 times lower than in the striatum and are compatible with immunohistochemical findings, respectively [12,14,15,16]. The same was the case for other cholinergic markers [17,18].

### 2.2. Postnatal Development of Cholinergic Neurons

During postnatal development of mice or rats, their ChAT, VAChT and ChT1 levels/activities in the brain increase several-fold in respective subcortical and cortical regions containing either perikaryons or presynaptic terminals of cholinergic neurons, respectively [17,18,19,20,21]. The key developmental parameter for maturation of cholinergic neurons is a several-fold increase in content and evoked quantal ACh release in synaptic terminals and axonal varicosities [6,8,22,23].

That assures multidirectional development, structural enrichment and maintenance of cognitive and motor functions in prenatal and postnatal life of animals and humans. The nerve growth factor (NGF) and brain-derived neurotrophic factor (BDNF) through their TrkA and TrkB high-affinity, stimulatory and p75NTR low-affinity, suppressory receptors regulate expression, survival and growth, viability of the entire cholinergic system during embryonal and postnatal development as well as in the mature period [11,24,25,26]. Developmental pattern and subcellular localization of ^125^I-NGF binding sites in chick embryo brain correlated with alterations in activity of ChAT and other cholinergic markers [24]. In addition, deficient neurotrophic signaling through TrkA, TrkB, p75NTR and other neurotrophic receptors may generate multiple clinical symptoms of physical and mental retardation accompanied by metabolic and structural neurodegeneration [27,28,29]. On the other hand, boosting expression of cholinergic phenotype by cAMP and retinoic acid elevated surface p75NTR density, causing NGF to decrease ChAT activity [26,29]. That also sensitized cholinergic neurons to excitotoxic insults and Aβ load through retrograde apoptotic signaling [26,30]. Such a mechanism may play a regulatory role in embryonal and postnatal growth being detrimental in aging animals/people who suffer a number of cholinergic encephalopathies including Alzheimer’s disease, Wernicke–Korsakoff or Parkinson’s dementias [30,31,32].

### 2.3. Intraneuronal Distribution of ChAT

Differential and density gradient centrifugation homogenates from different brain regions provided information about intraneuronal compartmentalization of ACh transmitter metabolism. Thus, medulla oblongata or basal nuclei containing almost exclusively perikaryons of cholinergic neurons display high relative specific activity of ChAT, about 1.80–1.92 in subfraction S_3_ containing cytoplasm from cholinergic and noncholinergic neuronal and glial cell bodies, and no enrichment in synaptosomal subfraction B [12,15]. On the other hand, preferential localization of ChAT in subfraction B with relative specific activity (RSA) 1.78–2.12 was seen in hippocampus, parietal cortex and striatum that are rich in cholinergic nerve terminals. On the contrary, low 0.39–0.60 values in S_3_ whole cytoplasmic fractions result from the low-content cholinergic neuronal somas [12,33]. These results correspond with immunohistochemical distribution for ChAT in cholinergic perikaryons and axonal terminals, in respective brain structures [34]. Hence, studies of regional and subcellular compartmentalization of ChAT/ACh and other cholinergic compounds enable quantitative assessment of distributional diversity-specific parts of cholinergic neurons [12,30,34].

## 3. Acetyl-CoA Metabolism in Cholinergic Neurons

### 3.1. Synthesis of Neuronal Acetyl-CoA

**Figure 1:**
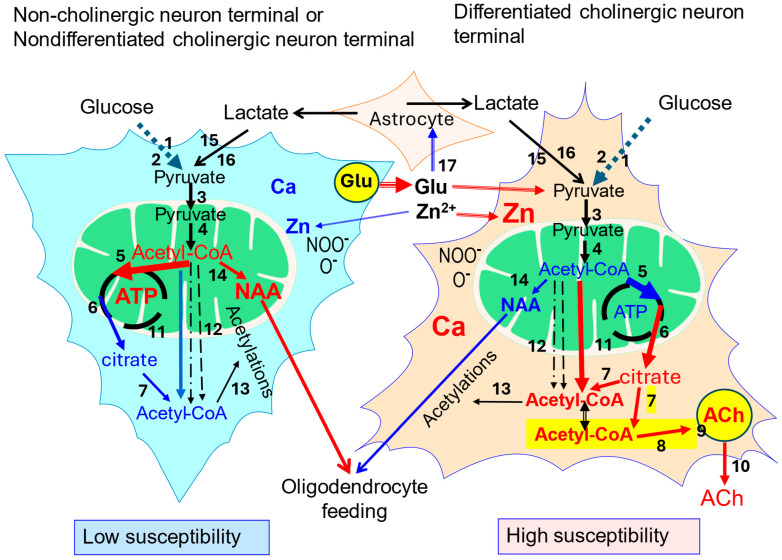
Comparison of acetyl-CoA metabolism in noncholinergic or low-cholinergic phenotype neurons and high-cholinergic phenotype neurons. Red color indicates higher rates or levels of given parameter against counterpart element; black numbers denote particular steps of acetyl-CoA/energy metabolism: 1—Glut 3; 2—glycolysis; 3—mitochondrial pyruvate carrier MPC1,2; 4—pyruvate dehydrogenase complex (PDHC); 5—citrate synthase (CS); 6—malate–citrate antiporter (SLC25A1); 7—ATP-citrate lyase (ACLY); 8—choline acetyltransferase (ChAT); yellow field—ACh synthesizing subcompartment; 9—vesicular acetylcholine transporter (VAChT); 10—quantal ACh release; 11—permeability transporter pores (PTPs); 12—carnitine acetyltransferase and acetyl hydrolase-synthase pathways, 13—diverse acetyltransferases; 14—N-aspartate-acetyl-CoA transferase (NAT); 15—monocarboxylate transporter MCT2 (SLC16A7); 16—lactate dehydrogenase (LDH); and 17—glutamate-Zn release and uptake by astrocytes. Yellow field marks cholinergic pool of acetyl-CoA linked with that of citrate–ACLY–citrate pathway coupled with ACh synthesis [6,8,10,21,39,44].

**Figure 2:**
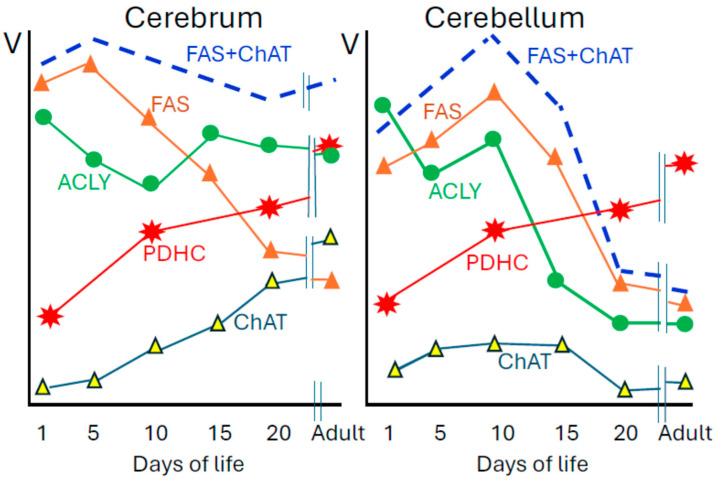
Developmental patterns of enzymes of acetyl-CoA metabolism in soluble S3 (FAS, ACLY, ChAT) and synaptosomal B fraction (PDHC) of rat forebrain and cerebellum. Specific activities of these enzymes in adult forebrain are as follows: PDHC 39.2; ACLY 6.9; FAS 0.0.91; ChAT 1.32 and in adult cerebellum: PDHC 37.7; ACLY 2.1; FAS 0.81; ChAT 0.14 nmol/min/mg protein. Adult rats are 100–120 days old. Data are taken from [16,19,20].

Acetyl-CoA is a key metabolite of the branching point between catabolic–intramitochondrial energy-producing tricarboxylic acid/respiratory chain cycles and multiple acetylation pathways taking place mainly in the cytoplasmic compartment but also in the endoplasmic reticulum, nucleus and mitochondria themselves [6]. The main precursor of acetyl-CoA in all types of brain cells is pyruvate-derived from glycolytic metabolism of glucose taking place in the cytoplasmic compartment. It is transported into mitochondria by the inner membrane located in mitochondrial pyruvate carrier MPC1,2 (Figure 1). In mitochondria pyruvate is converted to acetyl-CoA in the pyruvate dehydrogenase complex (PDHC) reaction. Glucose from the extracellular compartment is transported through the blood–brain barrier by a 55 kD GLUT1 transporter of high-capacity moderate-affinity constant of about 8 mM and density inversely regulated by glycemia [35,36]. The concentration of glucose in internal extracellular compartments of the brain is one third lower than in plasma. It is due to avid glucose utilization mainly by neuronal cells. Neurons express Glut3, high high-affinity glucose transporter on their plasma membranes of Km 2.5–2.8 mM, which may assure a relatively adequate supply of this metabolite under moderate hypoglycemia conditions (Figure 1). On the other hand, glial cells display 45 kD GLUT1 and in their glycolytic pathway synthetize surplus lactate against their own mitochondrial capacity for its oxidative utilization. Therefore, astrocytes and to a lesser degree oligodendrocytes release lactate to the extracellular space through low-affinity monocarboxylate transporter 4 (MCT4), to be taken up by neurons through their surface monocarboxylate high-affinity MCT2 (Figure 1) [37,38,39]. This pool of lactate through LDH and PDHC reactions may supply complementary acetyl units for neuronal TCA, particularly in hypoglycemic conditions [35,36]. It is estimated that, at an upper physiological extracellular concentration, about 1.0 mM lactate may substitute about 10% glycolysis in acetyl-CoA synthesis and energy production. Such replacement may rise up to 25% during heavy 10 mM lactic acidemia [39,40]. The TCA cycle of the entire neuronal pool, irrespective of transmitter system, consumes about 90% of acetyl-CoA produced by PDHC for obligatory energy (ATP) production indispensable for restoring resting neuronal plasma membrane potentials after each depolarization cycle. It is facilitated by very high activity of citrate synthase with high affinities to acetyl-CoA (Km = 4.8µM) and oxaloacetate (Km −3.0 µM) and reaction equilibrium shifted far toward citrate (K 1.01 × 10^6^) [41,42]. About 1–3% synthesized acetyl-CoA is used by mitochondrial aspartate N-acetyltransferase for synthesis of N-acetyl-aspartate (NAA), which is indispensable for feeding oligodendrocytes with acetyl units necessary for lipid synthesis forming myelin sheets (Figure 1 and Figure 2) [43,44].

### 3.2. Extramitochondrial Neuronal Acetyl-CoA

The remaining, nearly 10% fraction of synthesized acetyl-CoA is transported out of mitochondria and used for hundreds of acetylation reactions catalyzed by specific acetyltransferases located in the cytoplasm, endoplasmic reticulum and nucleus. They acetylate amino acids, coenzymes, carboxylic acids and amines yielding products of new biological activities such as N-acetyl-aspartate (NAA) in neuronal mitochondria, acetyl-serotonin in cytoplasm or acetylated histones in nucleus [6,44]. NAA is a principal acetylated amino acid in the brain of about 10 mM concentration. It is synthesized exclusively in neuronal mitochondria by specific aspartate N-acetyltransferase (NAT) [44]. Alterations in acetylations of nuclear histones significantly affect transcriptional and translational properties of all brain cells including cholinergic ones [43,45]. There are no comparative studies on the correlation between NAT and ChAT distribution in the brain suggesting putative links between NAA and the cholinergic compartment of acetyl moiety [44]. On the contrary, an increase in cholinergic phenotype in SN56 was accompanied by a decrease in their NAA content despite elevation of NAT [10]. It may be explained by differentiation-evoked translocation of acetyl-CoA from the mitochondrial to cytoplasmic compartment causing significant decrease in its level in mitochondria [6,46]. Kinetic studies of NAT revealed that in vivo it may strongly depend on acetyl-CoA concentration as its Km of 58 µM is about five times higher than this substrate level in the brain equal to 12 µM [46]. The overall acetyl-CoA-consuming capacity of these acetylating reactions remains unknown. However, they are important for neuronal functions on metabolic, proteomic and genomic levels. These diverse reactions consume a significant part of the remaining 10% fraction of the acetyl-CoA pool. In cholinergic neurons for this synthetic pool of acetyl-CoA additionally competes with ChAT, which utilizes acetyl units for synthesis and the maintenance stable level of vesicular, releasable quantal pool of ACh. It is estimated that ACh synthesis consumes about 3% of the overall pyruvate-derived mitochondrial acetyl-CoA pool in the neuronal compartment [46,47]. That, however, may correspond to up to 30–60% part of acetyl-CoA metabolic fluxes inside the cytoplasmic compartment, not linked directly with obligatory energy production (Figure 1). Therefore, disturbances in brain acetyl-CoA metabolism exert much stronger impact on cholinergic neurons’ wellbeing than on non-cholinergic ones [6,26,48]. In fact, inhibition enzymes of acetyl-CoA metabolism by several common cytotoxic conditions such as excitotoxic Zn^2+^, Al-excess, or thiamine deficiency brought about much greater loss of viability of highly differentiated than non-differentiated SN56 cholinergic neuronal cells (DCNs). These alterations correlated well with decreases of ChAT, ACh, ATP and acetyl-CoA in DCNs against none or minor ones in non-differentiated neurons (Figure 1) [46,49,50,51,52,53].

These data indicate that the provision of acetyl-CoA from nerve terminal mitochondria to the synaptoplasmic compartment may play a significant role in the regulation rate of ACh synthesis and cholinergic signaling. It also results from the fact that acetyl-CoA Km for ChAT is several times higher than its concentration in synaptoplasm. Therefore, alterations in acetyl-CoA availability in this compartment alone may significantly affect the rate of this reaction and size of the releasable ACh pool [51]. For this reason, direct or indirect assessment of the “cholinergic” pool of acetyl-CoA could be important for understanding disturbances in mechanisms of different cholinergic encephalopathies (Figure 1).

### 3.3. PDHC and Cholinergic Neurons

Intramitochondrial PDHC reaction is the main source of acetyl-CoA in all neuronal and glial brain cells. One may expect that cholinergic neurons due to their specificity should display higher levels of PDHC. However, patterns of ChAT, TrkA. VAChT in nerve terminals isolated from regions of different density cholinergic neurons do not correlate with the distribution of PDHC activity, specific proteins and mRNA [12,52]. The expression of the E1 subunit or PDHC activity in cerebellum containing very low content of cholinergic neurons among all brain macrostructures was, respectively, higher or similar to that in cholinergic-rich regions [12,52,54]. Also activities of other mitochondrial, acetyl-CoA-synthesizing enzymes such as carnitine acetyltransferase and acetyl-CoA synthase showed no correlation with the abundance of cholinergic neurons in a number of regions [19]. In accord with those findings, differentiation of clonal cholinergic septal SN56 neuronal cells caused no change in PDHC activity despite a several-fold increase in ChAT activity and ACh synthesis (Figure 2) [8,16]. Also 2–3 fold increases in PDHC activity over the course of postnatal maturation of the rat brains are similar in all regions due to maturation of whole neuronal and glial populations, irrespective of abundance of cholinergic innervation (Figure 2) [12,54]. At the same time ChAT activities and utilization of acetyl-CoA for ACh synthesis in the cortex increase 11–15 times [12,19]. These data indicate that developmental alterations in neuronal PDHC are directed by mechanisms independent of those regulating expression of cholinergic locus. Hence, efficiency of pyruvate conversion to acetyl-CoA may become a critical point balancing mature cholinergic neurons’ viability and high rates of cholinergic neurotransmission [6,51]. Also, even regional distribution of PDHC in nerve terminals points to similar sizes of primary pools of acetyl-CoA generated in neuronal mitochondrial compartments of different transmitter systems [51,54]. Decreases in brain PDHC and other enzymes of acetyl-CoA–energy metabolism were observed in humans suffering from Alzheimer’s or Parkinson’s diseases, thiamine pyrophosphate deficiency and alcoholics or prevalent vascular encephalopathies [55,56,57]. Similar alterations take place in animal models of cholinergic encephalopathies. In different transgenic mouse models of Alzheimer’s disease, loss of cognitive functions correlated with Aβ or tau overload in the brain. However, loss of cholinergic neuron density and their localization as well as disturbances in ACh/acetyl-CoA-linked metabolic pathways were combined in a very diverse manner [58,59]. Some of them revealed disturbances in energy metabolism accompanying cholinergic deficits [58]. Sixteen-month-old AβPP-Tg2576 mice displayed cognitive deficits and significant Aβ load in the brain, but there was no suppression of PDHC, KDHC, ACLY or ChAT activities nor cholinergic neuron density in immunohistochemistry [53,58]. However, in isolated nerve terminals, pyruvate utilization, mitochondrial and cytoplasmic acetyl-CoA, ACh content and release were reduced by about 30%. On the other hand, no enzymatic and metabolic alterations were found in whole-brain mitochondria [58,60]. These data indicate that functional and structural integrity of neuronal bodies may be retained despite marked loss of cognitive functions and inhibition of acetyl-CoA metabolic fluxes in nerve terminals [53,58]. Other diverse Tg animal models of AD provided concordant data on accumulation of Aβ and tau that were accompanied by cognitive failure and inhibition of ACh metabolism [61]. However, no information was found concerning acetyl-CoA in the brains of these Tg animals.

## 4. Cholinergic Neurons and ATP-Citrate Lyase

Mitochondrial acetyl-CoA is almost exclusively a precursor of cytoplasmic/synaptoplasmic acetyl-CoA necessary for several synthetic pathways. However, in resting conditions mitochondrial membrane is impermeable to acetyl-CoA and other CoA derivatives. Therefore, acetyl units are transported to the cytoplasm indirectly after conversion by citrate synthase (CS) to citrate [62]. This compound is easily transported to the cytoplasm by SLC25A1 malate–citrate antiporter (Figure 1) [62,63]. Nevertheless, citrate efflux to synaptoplasm is high enough to feed ACLY reaction, which converts it back to acetyl-CoA [64,65,66]. However, chronic hyperglycemia and ketonemia brought about increased pyruvate and acetoacetate utilization in brain nerve terminals of diabetic rats [67]. That resulted in a rise in citrate accumulation and acetyl-CoA level as well as ACh release in brain synaptosomes, respectively [67]. Accordingly, Tg mice with overexpression of SLC25A1 mitochondrial monocarboxylic acid transporter displayed increased levels of citrate and acetyl-CoA [68]. However, neither citrate synthase nor SLC25A1 displayed preferential localization in the cholinergic compartment, being highest in noncholinergic cerebellum [16,20,63,69]. Other pathways of indirect acetyl-CoA transport from mitochondria such as acetyl-CoA hydrolase, acetyl-CoA synthase and carnitine acetyltransferase are very active in liver, adipose tissue and muscle cells, but they do not significantly contribute to providing acetyl units for ACh synthesis under physiological conditions. In accord with those findings, (-)hydroxycitrate, a strong and specific inhibitor (Ki = 0.8 µM) for ACLY, was found to inhibit ACh synthesis but not more than 50% [64,66,70,71,72]. These findings leave the gap for an alternative direct transmembrane transport pathway of acetyl-CoA to synaptoplasm (Figure 1). Our data indicate that in K^+^-depolarized nerve terminals or cholinergic SN56 cells acetyl-CoA may be transported directly through Ca^2+^-induced PTP [73,74,75,76]. Accordingly, depolarization of differentiated SN56 cholinergic cells brought about Ca-influx and release from endoplasmic reticulum, along with a decrease in intramitochondrial and increase in cytoplasmic acetyl-CoA accompanied by an increase in ACh synthesis [48,73,74,75]. This phenomenon was reduced in low [Ca^2+^] medium, in the presence of Ca-channel blockers as well as in nondifferentiated SN56 cells with low activities of ChAT and ACh synthesis [48,75,76]. Such a mechanism may facilitate resynthesis of the releasable ACh pool during the repolarization phase of action potentials.

One can expect that cholinergic neurons/terminals may present a specific structural feature that would adapt them to efficient provision of acetyl units to the cytoplasmic site of ACh synthesis. Indeed, nerve ending subfractions B [33] from hippocampus or striatum, which contain 10 and 30% fractions of cholinergic synaptosomes displayed 4–5 times higher activity of ACLY than cerebellar ones with 1% cholinergic elements, respectively (Figure 2). These values perfectly correlated (r = 0.99, *p*, 0.0001) with respective activities of ChAT being 10 and 22 higher than those in cerebellum, respectively [12]. During postnatal development, activity of ChAT in the cortex increased 14 times but in the cerebellum remained at a low level of 0.1 nmol/min/mg protein. Maturation-linked decline of myelinization rate was accompanied by a similar rate, about 65% diminution of fatty acid synthetase activity. On the other hand, ACLY linked with fatty acid–lipid synthesis in the cortex displayed no change but 70% decrease in the cerebellum (Figure 1) [15,16,19]. Also electrolytic ablation of medial septum caused loss of 79% ChAT, 33% ACLY but no alterations in PDHC, CS and CaAT activities in synaptosomes of rat hippocampus [77]. Similar conclusion on preferential localization of ACLY in cholinergic neurons is drawn from intraventricular injection of 192IgG-saporin, which caused cholinergic neuron immunolesions in the cortex. That brought about 87 and 73% suppressions of ChAT and quantal ACh release and 32% inhibition of ACLY but no change in PDHC activity in fraction B, respectively [78]. Extrapolation of these data to 100% content of cholinergic neurons in the sample yields ACLY activity in cholinergic neurons as high as 20–40 nmols/min mg protein. Also ACLY Km values for citrate (0.13 mM), ATP (0.4 mM) and CoA-SH (0.0007 mM) are several times lower than their concentrations in the brain [79]. Thus, at adequate citrate efflux from mitochondria, ACLY may not be a limiting-regulatory step for provision of acetyl-CoA to the site of ACh synthesis. Tight developmental and regional correlations of ACLY with ChAT indicate its preferential co-localization in brain cholinergic neuron cytoplasm with formation-dedicated citrate-derived cholinergic pool of acetyl-CoA [5,6]. Experiments with a specific strong competitive inhibitor of ACLY (-)hydroxycitrate revealed that contribution of the ACLY pathway to ACh synthesis may vary regionally from 17% in hippocampus to 30 and 55% in nucleus caudatus and septum, respectively [64,65,70,71]. These data remain in accord with other findings on regional metabolic diversity of cholinergic neurons. They report the existence of differential regional distribution of ChAT immunoreactivities not correlating with random patterns of MCT, calbindin, calcitonin gene-related peptides and cholecystokinine in different basal nuclei and striatal regions taking part in cognitive functions [80,81].

## 5. Motor Cholinergic Neurons

Except cognition-managed by basal nuclei cholinergic neurons, other brain parts such as the stem, medulla oblongata and spinal cord contain diverse nuclei containing perikaryons of cholinergic motor neurons. All voluntary movements remain under brain control. Motor pathways begin in upper motor neurons located within the motor area in the rear cortex of the frontal lobe (areas 4 and 6), in which particular parts of the body are represented in an ordered manner. They are glutamatergic neurons, which receive various kinds of signals from sensory and other parts of the brain cortex as well as from the cerebellum converted to voluntary movements. From the cortex, signals are conveyed by axons to lower cholinergic motor neurons in the brain stem, medulla oblongata and anterior horns of spinal cord segments, where they form glutamatergic-excitatory synapses on perikaryons of cholinergic motor neurons. Those project their myelinated axons out of the central nervous system into motor nerves to respective striated muscles. Every single axon forms about 150 neuro-muscular junctions on muscle fibers [8,82]. All lower motor neurons are cholinergic. Depolarization waves from perikaryons, through axons reach terminals constituting presynaptic parts of neuro-muscular junctions. It evokes Ca-dependent quantal release of vesicular ACh to the synaptic cleft. ACh binding to postsynaptic nicotinic cholinergic receptors opens their sodium channels yielding depolarization of myocytes and their Ca-dependent contraction of the sarcomere units [8,82]. The capacity for ACh synthesis and release in the cholinergic motor neurons is proportional to the activity of ChAT, which is several times higher than in cognition-forming ones [12,16,83,84]. It is compatible with a very wide range of alterations in frequency, intensity and duration of voluntary movements of particular muscles. In such conditions the maintenance of a stable quantal pool of ACh is crucial for neuro-muscular unit function. For instance, the activities of ChAT in cholinergic motor nuclei of cranial nerves (*nucleus motorius n. facialis* or *n. hyoglossus*) appeared to be 10–30 times higher than those bearing cognitive functions (reticular formation, raphe nuclei) [83]. Also other cholinergic markers such as acetylcholinesterase or [^3^H]Quinuclidinyl benzilate ([^3^H]QNB-muscarinic receptor antagonist), displayed higher expression in brain stem and spinal cord motor nuclei than in sensory regions, when studied by radiochemical and enzymatic methods [84]. They also correlated with distribution of ChAT in discrete areas of the spinal cord [84]. Note that ChAT is synthesized only in perikaryons and has to undergo long axonal transport to reach the site of synthesis transmitter pool of ACh in presynaptic parts of end plates. The injury of motor nerves has served as a model for studies of axonal transport for about 100 years [85,86]. They invariably reveal that activities/levels of ChAT/ACh/VAChT decrease on both sides of the cut nerve early after intervention, and they tend to return toward normal values in favorable conditions [87,88]. One of them is adequate provision of energy by glycolysis and TCA. Proper activity of PDHC may be crucial for survival and structural/functional recovery of axons after crush injury or other noxious conditions, as most of the rat sciatic nerves caused upregulation of the E2 subunit of PDHC in the early posttraumatic period, thereby promoting its regeneration. Knock down of the E2 gene inhibited axonal posttraumatic outgrowth [89].

There is no direct data on acetyl-CoA vs. ACh synthesis interactions in lower motor neuron compartments. However, some indirect data indicate that they may not be interdependent. Thus activity of PDHC in mitochondria from different brain regions displayed no correlation with abundance of cholinergic structures [12,16]. Moreover, assaying ChAT in single motor neurons isolated from anterior horn nuclei revealed the presence of twenty-fold differences in enzyme activities [90]. That suggests the existence of axonal end plate-specific mechanisms for a wide range of adaptive alterations in acetyl-CoA and ACh synthesis/quantal release matching with variable physical effort conditions. Transport of full-spectrum proteins, whole mitochondria synthesized in perikaryon and accompanying metabolites by axons’ microtubular system requires continuous substrate supply to produce large amounts of energy [91,92]. Under normal conditions enzyme levels/activities (ChAT) and metabolites including ACh, ATP were similar both in proximal and distal parts of motor neurons indicating existence of efficient transport-homeostatic mechanisms [85,87,93]. Oligodendrocytes through their myelin sheaths cover motor peripheral axons, transport substrates such as glucose, lactate or amino acids to the periaxonal compartment on site. Axons take them up through MCT2 and GLUT3 transporters assuring proper rates of energy pathways, thereby maintaining ATP levels in each axon segment [94].

Thus, PDHC feeding the TCA cycle with acetyl-CoA plays an important role in the maintenance of motor axons. This was demonstrated on sciatic nerve axons, where pyruvate through PDHC reaction served both as a direct provider of acetyl-CoA for TCA in axonal mitochondria as well as a source of acetyl-CoA for a number of acetylation reactions in nucleus and endoplasmic reticulum. Crush injury of this nerve upregulates the E2 subunit of PDHC over the course of regeneration. Knocking down its gene restrains axon healing. That is probably dependent on nuclear subfraction of PDHC, which provides acetyl-CoA directly to histone transacetylase, which acetylates histone 3K9 and triggers a multistep pathway promoting axon regeneration [89]. The decrease in ATP increased axoplasmic viscosity, causing condensation of different neurodegeneration-linked proteins. These conditions could be prevented by nicotinamide mononucleotide treatment, suggesting that hydrotropic activity of ATP plays a crucial role in the regulation of axonal/neuronal homeostasis [95]. Equilibrium between mitochondrial and cytoplasmic acetyl-CoA pools seems to be also valid for motor neurons. Human motor neurons harboring mutation MT-ATP6, which impairs ATP-synthase, display metabolic compensation in the form of excessive utilization of acetyl-CoA in mitochondria yielding its deficit in the cytoplasmic compartment, suppressing histone acetylations and ACh synthesis [96]. Oxidative stress in *Drosophila melanogaster* cultured primary neurons, brought about inhibition of PDHC, succinate dehydrogenase, adenine nucleotide translocase, frataxin, and superoxide dismutase impaired axonal transport of mitochondria and microtubular decomposition. Loss of the remaining eight energy-linked proteins did not affect axonal transport [97].

Early findings demonstrating high activity of ACLY in the brain stem and medulla oblongata and their correlations with ChAT and ACLY activities suggest more preferential expression of this enzyme in the motor than in cognition-linked cholinergic neurons [15,16,83,84]. Hence, it may play a similar role as key supplier of acetyl-CoA for ACh synthesis in neuro-muscular junctions. In addition, the axonal level of ACLY is upregulated by binding with elongator complex (Elp3), which supplies acetyl-CoA directly to α-tubulin acetyltransferase (Atat-1), which acetylates the tubulin α-subunit promoting recruitment of kinesin and other molecular motors along microtubulin tracks [98]. Therefore, ACLY activates axonal transport in mice cortical neurons, fly larva motoneurons, *Drosophila melanogaster* and human fibroblasts. Knock down of Elp3 depressed the ACLY level. Accordingly overexpression of ACLY alleviated this inhibition increasing acetylation of α-tubulin. Knock down of *ATAT*-1 gene decreased tubulin acetylation and upset axonal transport. On the other hand, inhibition of ACLY with very high 10 mM concentration of (-)hydroxycitrate resulted in only 20% decrease in tubulin acetylation [98]. The source of this discrepancy remains unexplained. ACLY also contributes to other mechanisms of neuritogenesis. One of them is stimulating axonal transport cholinergic particles to motor terminals by ataxia-related protein (caytaxin/BNIP-H). This heterotetrameric complex after binding kinesin mediated intraneuronal/axonal transport of mitochondria to their terminals [99]. ACLY forms a complex with cyataxin, which synergistically recruits ChAT leading to enhanced synthesis and release of ACh, which through muscarinic autoreceptors promotes neurite outgrowth [100]. This property of ACLY is crucial in the embryonal and developmental period of life. Deficiency of BNIP-H abrogates development of motor neurons and manifests in humans as hereditary cerebellar ataxia or dystonia [100]. ACLY also takes part in activation of neural stem cells toward a mature phenotype by provision of acetyl-CoA for histone acetylations [101]. Axonal ACLY activity in this compartment is regulated by alterations of its endogenous inhibitor, D-2-hydroxyglutatrate. The latter is oxidized to 2-oxoglutarate by D-2-hydroxyglutarate dehydrogenase. Decrease in D-2-hydroxyglutarate alleviates ACLY inhibition, which provides more acetyl-CoA for histone acetylations, triggering stem cell activation [101,102]. These data demonstrate that ACLY is capable of forming diverse protein complexes, which indirectly support cholinergic neuronal structural and functional competence in the form of coherent voluntary neurotransmission.

## 6. Conclusions

Quantal ACh release from depolarization-activated cholinergic neurons/terminals triggers ChAT-out of equilibrium dependent resynthesis of transmitter pool, which apparently utilizes a significant fraction of the extra-mitochondrial non-catabolic pool of acetyl-CoA [47]. In fact, differentiation of SN56 caused a decrease in acetyl-CoA content in mitochondria and its rise in cytoplasm, which would be compatible with an elevated rate of ACh synthesis [46,48]. Those findings support the thesis that, in DCN, a greater fraction of acetyl-CoA was translocated from mitochondria to cytoplasm to match with elevated ACh release and resynthesis. It could be due to by 2-fold higher increase in Ca^2+^ accumulation in depolarized DCN, which activated PTP in mitochondrial membranes [46,48,103]. Similar effects were observed in SN56 differentiated with NGF, which displayed high intracellular Ca and overexpression of cell surface neuro-suppessory p75NTR [26,29,31]. In those conditions direct transport through PTP could provide a complementary pool of acetyl-CoA linked with ACh synthesis. On the other hand, it would make highly differentiated cholinergic neurons more prone to neurotoxic inputs [46,49,50]. They could be partially overcome by Ca-channel blockers and/or anti-p75NTR antibodies [48,76,104].

The majority of cholinergic encephalopathies is linked with energy failure on the PDHC step and/or other TCA cycle enzymes in mitochondria [55,56]. There are, however, small fractions of PDHC present in the endoplasmic reticulum and nucleus, which are linked with direct provision of acetyl-CoA for diverse proteins including histone transacetylases [105,106]. Traces of ACLY were also detected in these structures serving apparently as a complementary pathway supplying citrate-derived acetyl-CoA directly to structurally adjacent acetyltransferases [107]. However, no specific links with ACh metabolism were reported so far. On the other hand, ACLY, thanks to preferential localization in cholinergic neurons, may generate an additional pool of cytoplasmic acetyl-CoA dedicated specifically for ACh transmitter synthesis. Therefore, it may form in cholinergic nerve terminals, a specific cytoplasmic cholinergic domain that consists of ACLY-ChAT-VAChT, which stabilizes the releasable pool of ACh [18,21]. It is also capable of forming various complexes with diverse proteins contributing to development, maintenance and interneuronal interactions as well as with other cell types in the central and peripheral nervous system.

## Data Availability

No new data were created or analyzed in this study. Data sharing is not applicable to this article.

## Abbreviations

ACh—acetylcholine; ACLY—ATP-citrate-oxaloacetate lyase; ChAT—choline acetyltransferase; ChT1—high-affinity choline transporter; CS—citrate synthase; DCNs—differentiated cholinergic neuronal cells; FAS—fatty acid synthetase; LDH—lactate dehydrogenase; MCT2/SLC16A7—monocarboxylate transporter 2; MPC1,2—mitochondrial pyruvate carrier 1, 2; NAA—N-acetyl-aspartate; NAT—aspartate-N-acetyltransferase; NGF—nerve growth factor; PDHC—pyruvate dehydrogenase complex; PTP—permeability transition pore; [^3^H]QNB-^3^H—quinuclidinyl benzilate; SLC2A1—malate–citrate antiporter; and VAChT—vesicular acetylcholine transporter.
